# A community-based advanced nurse practitioner-led integrated oncology care model for adults receiving oral anticancer medication: a pilot study

**DOI:** 10.1186/s40814-024-01461-z

**Published:** 2024-02-29

**Authors:** Janice P. Richmond, Mary Grace Kelly, Alison Johnston, Patrick J. Murphy, Laura O’Connor, Paddy Gillespie, Anna Hobbins, Alberto Alvarez-Iglesias, Andrew W. Murphy

**Affiliations:** 1 Letterkenny University Hospital, Donegal, Ireland; 2 Discipline of General Practice, HRB Primary Care Clinical Trials Network Ireland, University of Galway, Galway, Ireland; 3 Centre for Research in Medical Devices (CÚRAM, RC/2073_P2) and Health Economics and Policy Analysis Centre, University of Galway, SFI 13, Galway, Ireland; 4 School of Medicine, HRB Clinical Research Facility, University of Galway, Galway, Ireland

**Keywords:** Cancer, Cancer care, Cancer management, Oral anticancer medications, Primary care, Advanced nurse practitioner, Feasibility study

## Abstract

Oral anti-cancer medications (OAMs) are being used increasingly within cancer care. OAMs offer the potential to improve patient convenience and increase hospital capacity. The clinical assessment for each cycle of OAMs requires specialist patient review often performed in hospital-based oncology units. Consequently, any potential improvement in patient expediency or increased hospital capacity that OAMs can offer is not realised. This study aimed to develop and pilot the specialist assessment of patients receiving OAMs by an Advanced Nurse Practitioner (ANP) in a community-based location.

The primary aim of this pilot study was to assess the feasibility of a community-based ANP-led integrated oncology care model for adults receiving OAMs in Ireland who met the pre-specified eligibility criteria. The objectives were to determine the feasibility of a definitive trial of this intervention by measuring patient safety, acceptability to patients and staff and cost of the new model of care.

This single-centre pilot study provided patient care (*n* = 37) to those receiving OAM therapies within a community setting for a 4-month period. Consent rate was high with no attrition other than for clinical reasons. There were 151 contacts with the sample during that time.

Results demonstrated that the ANP-led intervention and new model for OAM care was safe, highly acceptable to patients and staff and that related healthcare costs could be captured. Based on the success of this pilot study, the authors conclude that a community-based ANP-led integrated oncology care model for adults receiving OAMs is feasible, and a definitive trial is warranted.

**Trial registration** ISRCTN, ISRCTN10401455. Registered 30 November 2020.

**Supplementary Information:**

The online version contains supplementary material available at 10.1186/s40814-024-01461-z.

## Key messages regarding feasibility

What uncertainties existed regarding the feasibility?

- The primary aim of this pilot study was to assess the feasibility of a community-based ANP-led integrated oncology care model for adults receiving OAMs in Ireland.
- It was uncertain if this model of care would be acceptable to both participants and staff, and if recruitment and retention to a trial would be successful.
- It was also uncertain if patient safety could be maintained in a community-based location.
- Methods to assess interventions costs and outcomes were lacking.

What are the key feasibility findings?

- Adherence to the intervention among patients and staff was excellent, as were assessments of acceptability.
- Patient recruitment and retention to the pilot were close to 100% of those eligible for participation.
- Patient safety was successfully assessed and maintained throughout the pilot.
- Methods to assess intervention costs and outcomes were successfully implemented, and should be appropriate for a main trial.

What are the implications of the feasibility findings for the design of the main study?

- The implemented methods concerning recruitment, safety maintenance, cost and outcome assessment should be successful in a main trial.
- Aspects not assessed, including sample size calculations and the logistics of a multi-centre trial, will need careful attention.
- Given the success of this pilot, progression to a main trial is warranted.

## Background

The increasing prevalence of oral anti-cancer medications (OAMs) within cancer care has the potential to improve patient convenience and increase hospital capacity. OAMs have the same benefits and risks as systemic anti-cancer treatment (SACT) given intravenously in terms of tumour response (National Cancer Control Programme (NCCP) [19], treatment toxicities, potential for medication errors [19] and over or under adherence [10, 15, 22]. Due to these safety concerns, patients generally attend hospital-based oncology units for ongoing assessment and prescription of OAMs, [7, 11, 19, 17]. Consequently, any improvement in patient convenience or increase in hospital capacity that OAMs could offer has yet to be fully realised in Ireland.

This study aimed to develop and pilot a novel approach in Irish health care and move from hospital-based medical-led care to community-based advanced nurse practitioner (ANP)-led care for patients receiving OAMs. ANPs are senior, experienced nurses working in a specialism with advanced training including postgraduate training to at least Master’s academic level. This has enabled expansion of their scope allowing them to autonomously assess, physically examine, prescribe medication, request radiological imaging and plan care. This ANP-led study was proposed prior to the COVID-19 pandemic. It was due to commence when the pandemic was developing and consequently health care professionals (HCP) were forced to change practices to facilitate social distancing and hospital avoidance [32]. All this took place within the pre-existing situation of oncology services functioning at full capacity in Ireland [7]. This study was therefore not only relevant but was expedited due to COVID-19. In March 2020, the planned research site (a regional general hospital in Ireland) transferred the management of patients receiving OAMs from medical-led care in the hospital-based oncology unit to the care of the Oncology ANP.

This paper outlines the results of the pilot which is the second phase of a two-phase study and is based on the results of Phase 1, which of itself consisted of three components.

- Firstly, a scoping review informed this study and reported clinical practices for the monitoring of patients receiving OAM [23]. Overall, there was an identified paucity of international literature, yet a dedicated OAM clinic was endorsed with nurses and pharmacists identified as being of particular importance especially in education and ongoing management of patients receiving OAMs. Generally, care was hospital-based and no studies found OAM care in primary care/rural locations. Consequently, the pilot study reported in this current paper would be considered novel in healthcare, especially in Ireland.
- Subsequently, additional analysis of international guidelines around management of patients receiving anti-cancer medications identified recommendations for clinical practice. These recommendations were collated and from this best practice standards, clinical guideline (developed by the clinical authors to guide day-to-day clinical practice and ratified locally) and audit tools were developed [24]. Using this newly developed audit tool, a base-line audit was then performed [27] which measured care from the standards developed and demonstrated that the audit tools were fit for purpose.
- Thirdly, to determine the acceptability of ANP-led care and possible transition to an integrated context, a qualitative study was performed which involved all relevant stakeholders [25]. Analysis of the data using thematic analysis [4] generated four themes resulting in agreement that an integrated model of ANP-led care had significant benefits for patient care and the wider organisation of clinical oncology [25].

**Fig. 1:**
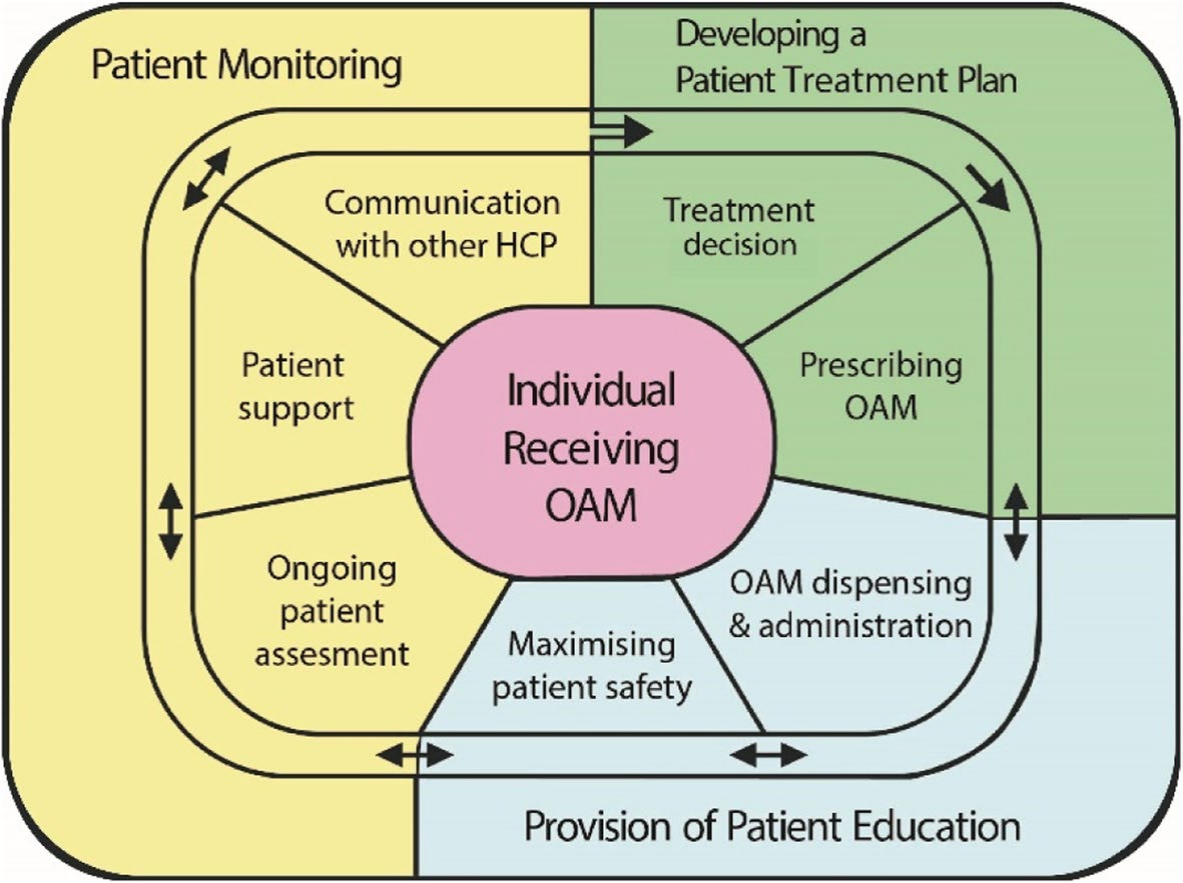
Model of care for an individual receiving OAM care [23]

The collective results of the three research activities in phase 1 listed above were presented by the study team to an advisory panel consisting of local and national experts who provided agreement to move to phase 2/pilot study (summary provided Appendix 1). This paper builds on previous work as outlined above, to pilot the newly developed model of care, the methodology of which is outlined in detail in an a priori protocol [26]. This current paper specifically outlines the pilot study of the new model of care and the intervention is the ANP performing ‘patient monitoring’ as portrayed in Fig. 1.

A stakeholder engaged approach was central to the entire study. Two public and patient involvement (PPI) contributors were research partners from project inception. The PPIs advised on language and information for the grant application and the Patient Information Leaflet required for the consent process. They participated in the stakeholders meeting and were invited to all 6-weekly research team interactions using Zoom application™.^1^ They were equal partners at all parts of the research process and any findings were presented to the entire team including the PPIs, in real-time, at the 6-weekly meetings. Specifically, PPIs reviewed all patient facing documentation and refined the themes in the qualitative analysis [25] which contributed directly to the development of the model.

### Aim and objectives of the current study

The pilot study aimed to assess the feasibility of a community-based ANP-led integrated oncology care model for adults receiving OAMs in Ireland. The primary objectives were to:

- Determine the feasibility of a definitive trial of this intervention focusing on patient safety, patient acceptability and cost of intervention.
- Determine staff acceptability for the new model of care.

A secondary objective was to identify feasibility issues to direct a future definitive trial.

## Methods

The methods described here are a summary of those in the published study protocol [26].

### Study setting

**Fig. 2:**
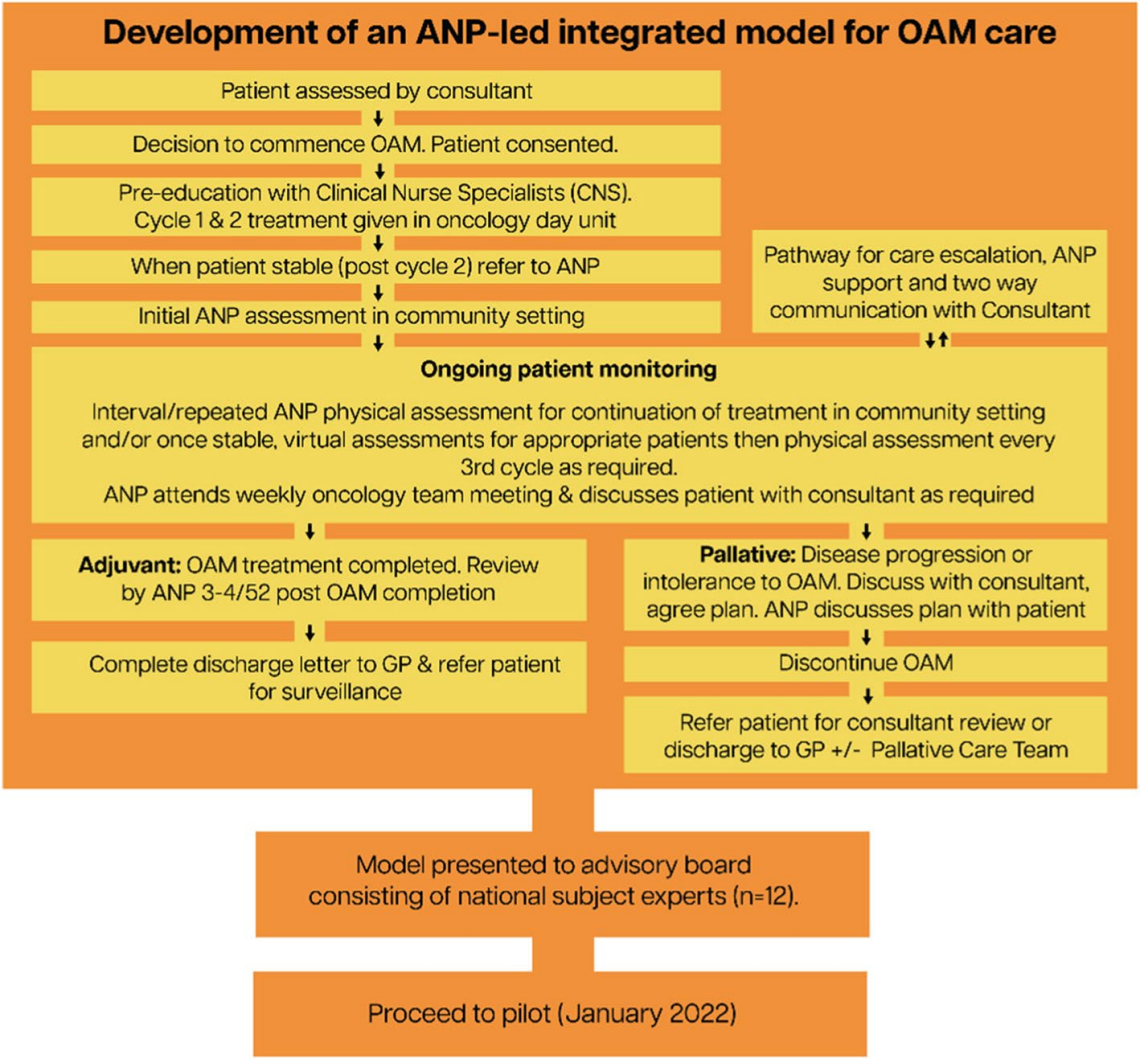
Development of an ANP-led integrated model for OAM care

This study assessed the feasibility of community-based ANP-led integrated oncology care model for adults receiving OAMs and to identify feasibility issues to direct a potential future definitive trial. The intervention of ‘patient monitoring’ (Fig. 1) consists of ongoing management of patient care following formal referral to the ANP once the treatment plan is developed in the hospital-based Outpatient Department, intensive patient education completed by the Clinical Nurse Specialists (CNS) and treatment initiated in the hospital-based oncology unit (Fig. 2).

This intervention for this pilot study was performed in a community-based location and involved continual communication with general practitioners (GPs) and other community staff while maintaining close links with the treating hospital-based oncology team. The study duration was 4 months, January 10th 2022 to May 9th 2022 inclusive. This was a single-centre study, with patient care provided both in-person and virtually. A clinical guideline to maximise patient safety and minimise risk had been developed by the clinical authors and was ratified locally prior to proceeding with the pilot.

### Eligibility criteria, participant recruitment and sample size

All eligible participants were recruited and consented from the cohort of patients being cared for by the ANP in the hospital-based OAM clinic. Eligible participants were over the age of 16, with solid tumours, had an Eastern Cooperative Oncology Group [21] performance status of 0–2, were cognitively able to provide consent and were receiving OAMs under the care of a consultant medical oncologist.

The cyclical nature of OAMs requires that patients are assessed on certain days of the week, reflective of the day they commenced treatment, which can also be determined by patients’ transport needs. The Health Service Executive community location had availability on two specific weekdays, therefore only patients whose assessments fell on those specific days were eligible to participate. A convenience sample of 67 participants was considered for the study (corresponding to the entire OAM clinical workload). Among those, 37 were deemed eligible, based on their availability on the days the intervention was set. For patients not enrolled in the study, provision of usual care continued as hospital-based care for the duration of the pilot. The protocol specified that a minimum of 100 assessments (either virtual or in-person) would be undertaken on the sample during the 4-month pilot phase.

### Primary outcomes

The primary outcomes for this study were to determine:

- Identification of any arising patient safety issues.
- Acceptability of the intervention among patients.
- Cost of intervention
- Acceptability of the intervention to staff.

### Data collection

#### Patient characteristics

A ‘Microsoft® Excel®’ spreadsheet was developed to capture gender, age and date of first assessment on the pilot study. Data were collected by the clinical authors (JPR and MGK), who reviewed the participants’ charts, extracted relevant data and completed the spreadsheet.

#### Cancer and cancer care characteristics

Similar to the data collection for participant characteristics, this data was obtained by the clinical authors reviewing the participants’ charts and completing the specifically developed Microsoft® Excel® spreadsheet. This chart review focused on primary cancer, years since diagnosis, specific OAM, cycle of OAM when enrolled on the pilot and whether the treatment had palliative or curative intent.

#### Patient safety

Patient safety in the community-based ANP-led OAM clinic was measured twofold. Firstly, there was a repetition of and comparison with, an initial audit performed in Phase 1 of this study [27] to determine adherence to best practice for patient assessments and OAM prescriptions. This best practice audit was conducted by a non-clinical member of the research team using specifically developed Microsoft® Excel® data capture tools [24] and involved retrospective chart reviews of randomly selected participant assessments (*n* = 20) and OAM prescriptions (*n* = 20) written by the ANP.

Secondly, data on the patient safety aspects of the intervention were captured in real-time by recording key safety measures on a Microsoft® Excel® tool specifically designed for this study [26]. This was performed at all clinics including identification of whether the participant was assessed on time, if laboratory samples were reserved at the appropriate interval as per the OAM drug protocol [20] and identification of any clinical incidents or near misses. Any ad hoc queries from community pharmacists were recorded as the OAM prescriptions should be completed with adequate information, and therefore any queries regarding dose/medication would be deemed a near miss. Any queries from GPs/community nurses were captured to determine the adequacy of communication with those care providers. Furthermore, any out-of-clinic questions from participants or family members were documented, providing an indication of any potential deficits in information-giving or education in the OAM clinic.

#### Patient acceptability

Acceptability of the intervention among patient participants was assessed using the 7-item EORTC-OUT-PATSAT7, a tool designed to capture the perceptions of patients with cancer regarding the service and organisation of care they receive [3]. Items are scored on a 5-point Likert scale ranging from 1 (‘poor’) to 5 (‘excellent’) with a higher score indicating acceptability of the intervention. The instrument includes 3 items assessing care convenience, 3 items assessing the transition of care, and 1 item assessing continuity of care.

#### Staff acceptability

Acceptability of the intervention among staff was assessed using an anonymous online questionnaire hosted on Microsoft® Forms. This questionnaire included 3 items developed by the authors: ‘1. Do you believe that patients receiving oral anti-cancer medications being assessed outside of the hospital setting is a good idea’, ‘2. Even if you were not directly involved in the care of these patients, we want to know how acceptable did you find this change of care?’, and ‘3. Are you supportive of the work of the ANP assessing patients receiving oral anti-cancer medications in a community setting continuing?’. All items were scored on a 5-point Likert scale ranging from 1 (low support) to 5 (high support). Respondents had the option to give the rationale for the scores they provided, and to give any further comments of interest. Finally, respondents had the option to request a separate confidential feedback interview with a member of the study team (author PJM).

#### Health economic analysis

The health economic analysis consisted of several related components. First, a cost analysis was undertaken to estimate the cost of implementing the intervention in clinical practice. This analysis was directly informed and guided by the process flow diagram, which was developed, presented and endorsed by the advisory panel of local and national experts at the end of Phase 1 of this study (Fig. 2). Data were collected on resource use related to all components of intervention delivery. Unit cost data, in 2021 € prices, were identified and applied to estimate the cost per-patient of implementing the proposed model of care. The costing included a range of resources such as staff time (consultant oncologist, ANP, CNS, pharmacist and administrator input), equipment, consumables, room rental, scans and tests. This data was recorded prospectively by the study research team. Where necessary, unit costs were transformed using the health component of the consumer price index from the Central Statistics Office [6], as per the Health Information and Quality Authority (HIQA) guidelines [13].

Second, data relating to the use of primary and secondary healthcare services over the course of the trial were captured at 4 months follow-up and a vector of unit costs was applied to calculate the costs associated with this resource activity (The EQ-5D-5L index score ranges from a minimum score of minus 0.974, representing the worst possible health state, to a maximum score of 1, representing the best possible health state; Table 1). Third, out-of-pocket expenses relating to patients’ time input, travel and parking costs at 4 months follow-up were estimated. Fourth, a preference-based health-related quality of life outcome, based on responses to the EuroQol EQ-5D-5L instrument was measured [2, 5, 9, 16, 28]. The EQ-5D-5L consists of five dimensions: mobility, self-care, usual activities, pain or discomfort and anxiety or depression [1]. Each dimension has five levels of severity: no problems, slight problems, moderate problems, severe problems or unable/extreme problems. EQ-5D-5L responses are transformed using an algorithm into a single health state index score, based on values elicited via the time trade-off and discrete choice approach for the Irish population [14]. The EQ-5D-5L index score ranges from a minimum score of -0.974, representing the worst possible health state, to a maximum score of 1, representing the best possible health state. The EQ-5D-5L has been generally accepted as suitable for use in oncology research [28].

**Table 1:** Categories of unit cost estimates in 2021 prices

Resource	Activity	Unit Cost €	Source
GP visits	Per Visit	€52	[30]
PN visits	Per Visit	€43	[30]
Outpatient visits	Per Visit	€142	HPO
*Other resources*			
Participant time	Per Day	€186.47	[6]
Participant travel	Per km	€0.38	[31]

*GP* general practitioner, *PN* practice nurse, *HPO* Healthcare Pricing Office Admitted Price List. Where necessary unit costs were inflated using the health component of the consumer price index from the Central Statistics Office as per HIQA guidelines

### Data analysis

Methodological issues were the central focus of this pilot, using the list of 14 methodological issues suggested to be examined in feasibility research [29]. The extension of CONSORT 2010 checklist specifically for pilot trials was used for reporting the study [8].

#### Data management and protection

A unique identifier number (UIN) was allocated to all the participants as they enrolled in the pilot and the UIN was known only to the two clinical authors directly involved in the participants’ care. This information was stored as a Microsoft® Excel® file in an encrypted computer in a locked single use office of the research nurse. All collected data were processed according to the General Data Protection Regulation requirements [12].

## Results

A summary of findings for the 14 methodological issues addressed in feasibility research are outlined in brief in Table 2.

**Table 2:** Summary of findings for the 14 methodological issues addressed in feasibility research

Methodological issue		Findings	Evidence
1	Did the feasibility/pilot study allow a sample size calculation for the main trial?	The pilot data provided valuable information related to the participant’s satisfaction and quality of care of the novel ANP-led integrated oncology setting. In the absence of an active comparator in the pilot trial, summaries of these instruments will be compared to equivalent summaries coming from patients receiving usual care, to inform the definitive trial of a sensible effect size to be considered in the sample size calculations	The EQ5D and the EORTC-OUT-PATSAT7 questionnaires were well accepted, demonstrated by high percentage of completion. Furthermore, the intervention in a future trial would be delivered in community-based clusters defined by the centres delivering the treatment. Through the involvement in the pilot study, and in communication with ANP-led oncology clinics similar to the one participating in the feasibility trial, members of the team obtained important information regarding the potential number of clusters and average cluster size (number of participants per cluster) for a future trial. This information will be very relevant when planning the sample size of the main cluster randomised clinical trial
2	What factors influenced eligibility and what proportion of those approached were eligible?	The major constraint on eligibility was the availability of the intervention setting, which was limited to 2 half-days per week All of those eligible were approached	Of the entire OAM clinic workload of 67 participants, 37 (55%) had assessments appropriate for days the intervention setting was available Of these 37, all (100%) were approached about participation
3	Was recruitment successful?	Yes, recruitment was successful	37 of 37 patients deemed eligible recruited
4	Did eligible participants consent?	Yes, all eligible participants consented	37 of 37 patients deemed eligible consented
5	Were participants successfully randomized and did randomization yield equality in groups?	This was a single-arm study (all participants received the intervention), and so randomisation was not relevant	Not applicable
6	Were blinding procedures adequate?	This was a single arm study (all participants received the intervention) and so blinding procedures were not relevant	Not applicable
7	Did participants adhere to the intervention?	Clinic attendance was high with no attrition other than completion of OAM course or disease progression on treatment Referrals by the wider multi-disciplinary team to the ANP continued during the study indicating that staff adhered to the referral process	152 assessments were performed on the 37 participants (101 face-to-face; 51 virtual). 2 assessments were missed by the patient as they had forgotten about the appointment, but they were rescheduled and attended with 7 days Staff referral of patients to the clinic matched usual care, at approximately 3 patients per month
8	Was the intervention acceptable to the participants?	Participants and staff both reacted positively to the intervention	Utilising the EORTC-OUT-PATSAT7 instrument, participants assessments of convenience, transition, and continuity of care were all at the top of the potential range, indicating very high levels of satisfaction with the intervention The 3 items rating staff acceptability of the intervention were all highly scored
9	Was it possible to calculate intervention costs and duration?	The methods developed and implemented for the health economics analysis proved feasible	See Table 7 for total intervention costs for the intervention, and Table 8 for patient resource use estimates at follow-up
10	Were outcome assessments completed?	Both participant and staff completion of outcome measures were very high	Full outcome measure data is available for 35 of the 37 participants (94.6%). Only 2 participants could not complete data collection due to disease progression/being unwell The staff satisfaction questionnaire was completed by 74.1% of staff members solicited (n = 23)
11	Were outcomes measured those that were the most appropriate outcomes?	Outcome measures were appropriate though high scores may suggest additional more sensitive measures may be required for a full trial	The EORTC-OUT-PATSAT7 instrument was deemed appropriate based on the clinical expertise of the research team, and the high completion rates suggest it was appropriate for participants in this study The EQ-5D-5L has been generally accepted as suitable for use in oncology research and no issues were encountered in the current study
12	Was retention to the study good?	All participants were retained within the study	No participants actively withdrew consent to participation. However, disease progression did impact upon data collection
13	Were the logistics of running a multicentre trial assessed?	This was a single-centre study, and so assessing the logistics of a multicentre trial was not possible	Not applicable
14	Did all components of the protocol work together?	The intervention components worked together as planned. Integrated care in a community location was provided to participants smoothly within existing oncology care	Judgement of the research team, combined with the lack of any problems with the intervention itself

### Issue 1: Did the study allow a sample size calculation for the main trial?

The data collected in this pilot provided valuable information related to the participants’ satisfaction with the quality of care received in the novel community-based ANP-led integrated model. Both the EORTC-OUT-PATSAT7 and the EQ-5D-5L questionnaires were accepted with high completion rates (see Issue 10 below). However, as this study did not include a control arm, it was not possible to estimate an effect size for the intervention, nor to calculate a sample size for a definitive trial. Please see the “Discussion” section “Sample Size Requirements for a Definitive Trial” section  for more on this issue.

### Issue 2: What factors influenced eligibility and what proportion of those approached were eligible?

As above (in section entitled Eligibility Criteria, Participant Recruitment and Sample Size), potential participants were recruited from the cohort of patients being cared for by the ANP in the hospital-based OAM clinic [26]. See above for a summary of eligibility criteria. For the 4-month duration of the study, this amounted to 67 participants. Also as mentioned above, a constraint on eligibility was the availability of the community-based intervention location, which was limited to 2 half-days per week and this constraint pertained to the entire study. Consequently, only patients whose assessments fell on those 2 half-days could potentially participate. This resulted in 37 participants being deemed eligible, amounting to 55% of the entire OAM clinic workload. Of these 37, all (100%) were approached about participation.

### Issue 3: Was recruitment successful?

Recruitment commenced in November 2021 and continued for the 4-month pre-defined duration of the study. All potentially eligible participants were recruited (*N* = 37, 100% of those eligible).

### Issue 4: Did eligible participants consent?

The ANP (author JPR) approached eligible patients. After the study was verbally explained, patients were given an information leaflet and a consent form to take home for consideration. The contact details of the research nurse (author MGK) were provided should they have any questions or wish to discuss the study further. The contact details of the research nurse were given to help distance the ANP from influencing the patient’s decision to participate. Signed consent forms were returned to the research nurse by mail using a prepaid envelope, or in-person to the ANP at a subsequent appointment. All eligible patients gave consent (*N* = 37). Descriptive statistics for characteristics of the consenting participants are provided in Table 3. Descriptive statistics for the characteristics of the cancers and cancer treatments for consenting participants are provided in Table 4.

**Table 3:** Characteristics of participants (*N* = 37)

Characteristics of participants	*n*
Years since diagnosis (Median(IQR)	3.5 years (2–8 years)
*Gender*	
Male	22
Female	15
*Ethnic/Cultural background*^a^	
Irish	30
European (Non Irish)	2
No response	4
Other	1
*Travel*	
Distance in kilometers to OAM assessments (Mean (SD))	38 km (± 21 km)
Use of car as transport to OAM assessments	37
*Cost of care*	
Medical Care Holder^a^^b^	26
GP visit card holder^a^	7
Health insurance holder^a^	11
Health insurance premium cost^a^	€1461.92 (+ / − €1265.49)
*Employment status*^a^	
Retired	19
Unemployed	4
Homemaker	3
Full-time employee	3
Part-time employee	2
Self-employed	2
Other	1
No response	1
Smoker^a^	6

^a^Data unavailable for 2 participants

^b^Medical card holders relates to anyone resident in Ireland with a weekly income below a government established figure for the family size and guarantees free oncology care and treatment

**Table 4:** Characteristics of cancer and cancer treatment for all participants (*N* = 37)

Characteristic of cancer and cancer treatment	*n*
Years since diagnosis (M (SD))	5.8 years (+ / − 6.3 years)
*Primary cancer*	
Breast	10
Gastrointestinal	5
Genitourinary	19
Lung	2
Other	1
*Type of OAM*	
Androgen biosynthesis inhibitor	13
Androgen receptor inhibitor	4
Anti-neoplastic/Cytotoxic medication	7
CDK4/6 Inhibitor	5
*Epidermal growth factor receptor (EGFR inhibitor)*	*2*
Kinase Inhibitor	3
Poly adenosine diphosphate-ribose polymerase (PARP) inhibitor	2
Vascular endothelial growth factor (VGEF) inhibitor	1
OAM cycle on first assessment in pilot study (Mean (SD))	Cycle 18 (+ / − 12 cycles)
*Aim of OAM treatment*	
Curative	7
Palliative	30

### Issue 5: Were participants successfully randomised and did randomisation yield equality in groups?

This was a single-arm study (all participants received the intervention), and so randomisation of participants was not relevant.

### Issue 6: Were blinding procedures adequate?

This was a single arm study (all participants received the intervention) and so blinding procedures were not relevant.

### Issue 7: Did participants adhere to the intervention?

#### Participant adherence

During the study, 152 assessments were performed on the 37 participants (101 in person to include physical examination; 51 virtual). During this time, two participants did not attend their appointment. When contacted by telephone they stated they had forgotten about the scheduled care; both had their care rescheduled and were assessed within 7 days. This does not represent non-adherence as both agreed to be rescheduled and continued participation in the study.

#### Staff adherence

Referrals received from the medical team to the community-based ANP-led oncology clinic were used to determine if staff were adherent to the intervention. During the 4-month study period, 10 new referrals were received. The referral rate in the 6-month period prior to study commencement was on average 3 per month. Therefore, the referral rate during the study period was consistent with usual practice.

### Issue 8: Was the intervention acceptable to participants?

#### Participant acceptability

As above, participant acceptability of the intervention was assessed using the 7-item EORTC-OUT-PATSAT7 instrument [3]. As shown in Table 5, scores for the 3 subscales of convenience, transition and continuity of care were all at the top of the potential range, indicating very high levels of satisfaction with the intervention.

**Table 5:** Assessments of participant acceptability with the intervention (*N* = 35)

EORTC-OUT-PATSAT7 Subscale	Potential range	Observed range	Mean	SD
Convenience	1–5	3–5	4.68	0.43
Transition	1–5	4–5	4.81	0.31
Continuity	1–5	4–5	4.86	0.36

#### Staff acceptability

The 3 items rating staff acceptability of the intervention were all highly scored (Table 6) with a staff response rate of 74.2% (*N* = 23). Ten staff respondents provided additional comments. These were reviewed by the research team and it was agreed that 7 praised the intervention and 3 offered helpful suggestions for consideration in continuing work (Appendix 2). There were no negative comments, and no uptake on the option of a separate confidential feedback interview.

**Table 6:** Assessments of staff acceptability of the intervention (*N* = 23)

Items	Potential range	Observed range	Mean	SD
Item 1: Merit	1–5	3–5	4.61	0.66
Item 2: Acceptability of change of care	1–5	3–5	4.61	0.72
Item 3: Support of continuation	1–5	2–5	4.70	0.77

### Issue 9: Was it possible to calculate intervention costs and duration?

The methods developed and implemented for the health economics analysis proved to be feasible. The total cost of the intervention implementation was estimated at €89,462, which resulted in a cost per-participant (*N* = 37) of €2418. This cost estimate is likely to fall if it proved feasible for additional patients to be facilitated by the community-based oncology clinic. The results from the process of costing the intervention implementation are presented in Table 7.

**Table 7:** Intervention costs for the ANP-led integrated care model (*N* = 37)

Resource item	Total	Total cost per participant
*Fixed costs*		
Equipment	€14,190	€384
*Variable costs*		
Room rental	€8,550	€231
Staff	€26,632	€720
Consumables	€22,301	€603
Scans	€12,249	€331
Tests	€5,540	€150
Intervention cost total	€89,462	€2,418

Price in 2021 Euro

The costs associated with the use of primary and secondary healthcare services over the 4-month follow up period are presented in Table 8, and reveal an average cost per participant of €431.80 (*SD* = €260.13).

**Table 8:** Resource use estimates at follow-up (*N* = 37)

Resource items	Usage (mean (SD))	Cost per participant in € (mean (SD))
GP Visits	1.17 (1.27)	€60.91 (66.11)
Practice Nurse Visits	1.17 (1.96)	€50.37 (84.40)
Outpatient Visits	2.26 (1.52)	€320.51 (216.02)
Total Healthcare Resource		€431.80 (260.13)

*GP* general practitioner. Completeness of data: 5% missing data for GP visits, practice nurse visits and outpatient visits

The results for out-of-pocket expenses show that on average, 6.67 (Median = 0.00; *SD* = 20.0) workdays were missed to attend appointments, and 0.63 (Median = 0.00; *SD* = 1.77) workdays were missed due to ill health over the 4-month follow-up period. The opportunity cost of this time was estimated at €1243.13 (Median = 0.00; *SD* = 3,729.40) to attend appointments and €116.54 (Median = 0.00; *SD* = 329.63) due to ill health, on average. In addition, those accompanying participants to appointments missed an average of 3.00 (Median = 3.00; *SD* = 1.15) workdays during the follow up period, resulting in an average cost of estimate of €559.41 (Median = 559.41; *SD* = 215.32). The average distance travelled was 40.41 km (Median = 40.00; *SD* = 27.48) for assessment appointments and 27.23 km (Median = 20.00; *SD* = 27.09) for blood tests, resulting in average travel cost estimates of €15.34 (Median = 15.18; *SD* = 10.43) for appointments and €10.33 (Median = 7.59; *SD* = 10.28) for blood tests. The average parking cost per appointment was €1.67 (Median = 1.50; *SD* = 0.76). In terms of preference-based health-related quality of life, the average EQ-5D-5L index score at 4-month follow-up was 0.84 (Median = 0.92; SD = 0.26), falling below the maximum score of 1 (Appendix 3).

### Issue 10: Were outcome assessments completed?

#### Outcomes related to patient safety

Data obtained from the repeat community-based audit of the randomly selected sample of 20 charts and OAM prescriptions, when compared to the hospital-based results completed in Phase 1 demonstrated an all-level improvement in all aspects of patient education, OAM prescribing and patient monitoring with adherence rates often reaching 100% and no deterioration in care detected [27]. The participant safety data obtained for all participants (*N* = 37) was compared to established parameters relating to the OAM drug protocols [20]. This comparison demonstrated that of the 152 assessments, 134 participants were reviewed as per protocol timing parameters. Thirteen assessments were performed at an earlier time than the OAM drug protocol required, due to providing imaging results (*n* = 12) or liquid biopsy results (*n* = 1) where relevant to their OAM treatment and required immediate communication with them. Four assessments were delayed beyond protocol standards and of these, 2 were delayed up to 3 weeks due to the participant recovering from COVID-19 (*n* = 1) or being on vacation (*n* = 1). A further 2 forgot about their appointment and were delayed by up to 1 week, when it was rescheduled.

Over the duration of the study, there were 31 queries to the ANP between appointments: 14 from participants, 6 from family members and 11 from a community HCP. The details of these are outlined in Table 9.

**Table 9:** Queries to ANP Oncology external to scheduled assessments

Origin of query	Number	Reason for query
Participants	14	• COVID related regarding symptoms/testing/isolation (8) • Informing ANP of date for radiology investigation (3) • Blood sampling timing (2) • Request for repeat OAM prescription (on investigation this had already been renewed) (1)
Family members	6	• Discussion of COVID status (4) • Request for update of in-patient’s care/status (2)
Community HCPs	11	• GPs planning care (10) • Dose clarification (steroids) (1)

Laboratory samples were reserved on average 11.6 h prior to the assessment. On comparison with the NCCP OAM protocols [20], this is an acceptable timeframe.

#### Patient-reported outcomes

Thirty-five of 37 participants completed the HEQs and the EORTC-OUT-PATSAT7 instrument. Complete data were provided for all 35 EORTC-OUT-PATSAT7 assessments. Almost all returned HEQs included some non-responses to items mostly relating to aspects of life that may not have been relevant to them(e.g. older people not having childcare costs to report), but there is no data to support this assertion. Of the 2 participants who did not complete the HEQ and the EORTC-OUT-PATSAT7, 1 died, and 1 became too unwell to participate in data collection.

#### Staff-reported outcomes

A link to the online questionnaire was sent via email or text to 31 oncology HCPs (3 medical oncologists, 2 registrars, 3 pharmacists, 4 nurse managers, 8 nurse specialists and 11 staff nurses). The response rate was 23/31 (74.2%); there was no staff uptake of the confidential feedback interview.

### Issue 11: Were outcomes measured those that were the most appropriate outcomes?

The EORTC-OUT-PATSAT7 instrument which addresses the perception of quality of aspects of care specific to ambulatory cancer care settings was deemed appropriate based on the clinical expertise of the research team; the high completion rates suggest it was appropriate for participants in this study and no issues were encountered in the current study.

### Issue 12: Was retention to the study good?

No participants actively withdrew consent to participation. Of the 37 enrolled on the study, 29 were continuing with their OAM treatment at the end of the study period (Appendix 4). Throughout the duration of the pilot, 2 had completed their OAM treatment course and a further 1 chose to discontinue treatment due to intolerance of toxicities. Five had their OAM discontinued permanently due to disease progression and one of these participants died. These data would be expected within a cohort of participants with cancer, especially as 81% were receiving palliative treatment. The findings represent the changing nature of cancer and the toxicities of treatment, rather than retention to the study itself.

### Issue 13: Were the logistics of running a multicentre trial assessed?

This was a single-centre study, and so assessing the logistics of a multicentre trial was not relevant.

### Issue 14: Did all components of the protocol work together?

The intervention components worked together as planned. Integrated care in a community-based location was provided to participants smoothly within existing oncology care.

## Discussion

This pilot study assessed the feasibility of a community-based ANP-led integrated oncology care model for adults receiving OAMs. The results indicated that this model of care was acceptable to both participants and staff, that patient safety could be assessed and maintained, and that related healthcare costs could be captured. Progression to a definitive trial is warranted which should include a control arm and investigate community-based ANP-led care versus hospital-based OAM care. The design, methodology and outcomes utilised in the pilot study could then be used/measured in the definitive trial.

### Implications for patient safety in a definitive trial

With any healthcare intervention, patient safety is key. This pilot recorded no clinical incidents or near misses, which are the baseline determinants of safe care. Furthermore, according to the NCCP OAM drug protocols (2022), all safety parameters in relation to patient monitoring were adhered to in full, including appropriate reserving of laboratory samples, timing/frequency of reviews and correct OAM prescribing practices. We therefore conclude that the methods developed to monitor and maintain patient safety in this pilot should be appropriate for a definitive trial.

### Implications for patient recruitment and retention in a definitive trial

We have shown above that the new model of care and the study procedures were acceptable to participants, as evidenced by excellent recruitment and retention rates. We acknowledge that recruitment by the caring ANP is not ideal. We attempted to mitigate this through the use of the research nurse. In a full trial, it may be preferable, resources permitting, to have the full consent process managed by a research nurse.

Our prior qualitative study found that patients have a desire for the same HCP to assess them at each clinic visit [25], something guaranteed in the community-based ANP-led clinic. In addition, as participants received their first 2 cycles of OAM treatment in the hospital-based oncology unit, they had already experienced exceptionally long wait times of 3–4 h (most of which was waiting for blood results and/or to be reviewed by a doctor), compared to approximately 1-7 min of a waiting time in the ANP-led clinic. Reduced waiting times were particularly important to patients during the COVID-19 pandemic. Participants were therefore cognizant of the improvements already offered in the ANP-led clinic, and expressed a desire to contribute to further developments in care. Finally, we note that the burden of participation was low, limited to the completion of the satisfaction measure and the HEQ. All of these factors combined contributed to the excellent retention rates. We believe that the same factors which produced excellent recruitment and retention rates in this pilot study will also produce excellent recruitment and retention rates in a multicentre definitive trial.

### Implications for staff support for a definitive trial

As with participants, we have shown that the new model of care and the study procedures were acceptable to clinical staff, as evidenced by referral rates and responses to the staff questionnaire. We believe however that acceptability among clinical staff was influenced by the knowledge that significant preparatory investigations had been conducted [23–25, 27], a detailed protocol had been developed [26], and most critically the community-based care was delivered by an experienced ANP. For a definitive multicentre trial to be successful, clinical staff will need to be assured that funding are available to set up the community-based clinics, and that sufficient clinical input from an experienced ANP is guaranteed.

### Implications for cost assessment in a definitive trial

As above, the methods developed for the health economics analysis proved to be feasible. Although patient assessment for OAM care is contact intensive (approx. 45 min/patient), the indicated cost per-patient in this pilot is likely to be an overestimate. A definitive trial would allow fixed costs to be spread over a larger number of participants, resulting in a lower cost per-patient. Also, the reported variable costs in this pilot were inclusive of radiological investigations and laboratory results for each OAM cycle, which would also be incurred in hospital-based oncology unit.

As mentioned above, a multicentre definitive trial would require significant input from experienced ANPs, and funding to allow set-up of the community-based OAM clinics. There may be greater costs arising from travel time for ANPs, particularly if ANPs were to provide community-based assessments in more than 1 location. Also, scheduling of assessments may become more onerous, requiring greater clinical input and therefore greater costs. These potential costs will require careful monitoring.

### Sample size requirements for a definitive trial

As above, since this study did not include a control arm, and summary data for the primary outcome in the usual care/control group was not available from the literature, it was not possible to estimate an effect size for the intervention, nor to calculate a sample size for a definitive trial. To calculate a sample size, further pre-trial data would need to be collected on the relevant outcomes for patients receiving usual care in the hospital-based oncology unit. Although outside the scope of the current study, this data collection should have low cost and resource implications. Furthermore, the intervention in the definitive trial will be delivered in community-based clusters defined by the hospital-based cancer centres delivering ANP-led treatment. Data related to the number of these clusters and the average cluster size (number of participants per cluster) are readily available.

### Limitations

The data in this pilot were obtained from a single site, and from participants in a single ANP’s workload. It is possible that the data are not representative of other hospitals, or other ANPs’ workloads. The participants characteristics (Table 3) and their cancers (Table 4) do appear representative of ANP workload in Ireland. While caution is always warranted, we believe transparency in reporting means the presented implications for a definitive trial are realistic.

Randomisation and blinding procedures were not relevant in the current study as all participants received the intervention. There was no control arm present in this study which is acknowledged as a limitation. A control arm, randomisation, and blinding procedures will require careful attention in a definitive trial to minimise relevant biases in the assessment of intervention outcomes and costs.

Comparison data and accepted clinical cut-offs to aid interpretation are lacking in our study due to the newness of the EORTC-OUT-PATSAT7 instrument. A Phase III study was completed and currently a Phase IV^2^ psychometric study is underway to further validate the measure, which will address this deficit [3].

For any multicentre trial offering OAM care in community locations, electronic patient records are the ideal. At the time of writing, electronic records are in the process of being incrementally introduced in Ireland for SACT care in the hospital-based oncology units and could be adopted for use in OAM clinics in the community prospectively.

A methodological limitation of the current study was the lack of pre-specified progression criteria. Pre-specified progression criteria assist decision-makers to judge whether, or how, to proceed to a future definitive trial [8]. A review by Mbuagbaw et al. [18] reported that only 1 in 5 protocols for randomised pilot trials included progression criteria. Those authors noted that ‘if progression criteria are not set a priori, there is a risk that some studies that did not do well in the pilot stage may be moved to a larger trial without modification or due acknowledgement of potential limitations’. We therefore acknowledge this limitation, but point to the transparency of our reporting and the unambiguous success of this pilot, and maintain that progression to a definitive trial is warranted.

A limitation of the health economic analysis was the limited number of healthcare resources included in the cost analysis. Future studies could include a wider range of resource activity to more fully capture the burden of care.

## Conclusion

This study was performed in one hospital, which serves a largely rural population. For national rollout, organisation of services and population demographics may vary, but the new model developed and piloted in this paper and the infrastructural requirements should broadly translate to any other service in Ireland. Based on the success of this pilot study, the results of which will be used to inform the design, methodology and instruments used to collect the data of a larger trial, we conclude that community-based ANP-led integrated oncology care model for adults receiving OAMs is feasible, and a definitive trial is warranted.

### Supplementary Information

**Supplementary material 1. **

## Data Availability

The data that support the findings of this study are available from authors J.R and A.M but restrictions apply to the availability of these data, which were used under license for the current study, and so are not publicly available. Data are however available from the authors upon reasonable request and with permission from J.R and A.M.

## Footnotes

1 ™ Zoom Video Communications, Inc. San Jose California 95,113, United States.

2 See EORTC guidelines for more details on phase III and phase 4 research available at: https://www.eortc.org/app/uploads/sites/2/2018/02/guidelines_for_developing_questionnaire-_final.pdf
