# Supplementary material for: A community-based advanced nurse practitioner-led integrated oncology care model for adults receiving oral anticancer medication: a pilot study
Source: Pilot Feasibility Stud. 2024 Feb 29;10:46. doi: 10.1186/s40814-024-01461-z (PMC10902979; doi:10.1186/s40814-024-01461-z)
Supplement: Supplementary file 1 — Supplementary material 1. [file 40814_2024_1461_MOESM1_ESM.docx]

# Appendix 1: Summary points agreed by advisory panel

Open discussion occurred and was minuted. Minutes were sent to advisory panel members for ratification. The key points are summarized below and color coded as follows:

- - Green-Being managed already and continue to use what’s in place.
  - Orange-Doable but needs further consideration.
  - Red-Pilot should answer this query.

Key points:

1. ANP-led OAM care and referral process working well and to continue with this within the pilot.
2. Blood taking and monitoring working already: continue this within the pilot but formalize governance within the guideline.
3. Current frequent communication with GPs and community pharmacists will continue.
4. Current process of assessment/admission of unwell patients will continue via acute oncology pathway.
5. Communication between consultant & ANP is vital and needs to continue within the pilot.
6. Reduction in patient travel through busy hospital should continue post COVID-19 within the pilot.
7. Guideline needs to be written and circulated to wider Multi-Disciplinary Team (MDT) (to include referral process and identify patient cohort, patient governance & management of the acutely unwell patient).
8. Guideline needs to be written and circulated to wider MDT team (to include referral process and identify patient cohort).
9. Consideration will be given as to how blood monitoring would work (and resources required) if care of these patients becomes fully integrated.
10. Communication of time critical issues is vital and needs addressed.
11. Monitor the MDT referrals and timelines.
12. National Cancer Information System (NCIS) which is a national electronic record system is critical to improving OAM prescription issues and is a prerequisite for truly integrated care and improving communication and co-ordination for truly integrated care.
13. Determine team requirements for true integrated care.
14. Further communication with oncology pharmacy and NCCP required to address these concerns which may transcend this project.

# Appendix 2: Free-text comments from the staff acceptability questionnaire.

- “The input of trained Oncology Pharmacists is important to support ANPs and Doctors in providing oral SACT in the community setting. Community Pharmacists don't have the necessary training.”
- “The critical factor for OAM patients is the specialist knowledge required in assessing them not the physical location. With the predicted doubling of cancer incidence and 66% expected increase in SACT delivery, necessary reform and alternative avenues such as this that are aligned with Sláintecare ^[[1]](#footnote-1)^ are critical.”
- “Oncology ANP are experienced nurses that have specialized knowledge of their field. Where the ANP deems suitable area to hold clinic is within this nurse’s specialist knowledge. Community clinics would be more suitable for the patient; the nurse would also have access to laboratory and radiology systems.”
- “Excellent pilot, proactive and beneficial for patients....looking forward to seeing the results of the pilot study.”
- “It's a great service that patients taking oral chemotherapy are well supported. Patients will feel more confident and comfortable that they are well monitored. Services like this will give patients more compliant.”
- “This takes patients out of a hospital setting and it also frees up the Day Unit.”
- “It takes patients away from a clinical area and maybe causes less anxiety to them, also Doctor's are available by phone for advice.”
- “The OAM initiative, heralded following the COVID-19 pandemic, has been a great benefit for the patient's experience and freed up space within the Day Unit. It has been invaluable to the processes and progress of the service.”
- “This is an excellent initiative as it allows for extra capacity within the Day Service setting and there is better continuity for the patients.”
- “Excellent pilot, looking forward to seeing it rolled out....an amazing initiative, well done to all those involved.”

**Appendix 3 - Proportion of responses by level of severity for EQ-5D-5L dimensions and index values at follow-up (n=37)**

| Dimensions | Levels | Baseline |
| --- | --- | --- |
|  |  | **N=37** |
|  |  | **%** |
| Mobility | |  |
|  | **None** | 68.57 |
|  | **Slight** | 17.14 |
|  | **Moderate** | 8.57 |
|  | **Severe** | 2.86 |
|  | **Unable** | 2.86 |
| Self-care | |  |
|  | **None** | 82.86 |
|  | **Slight** | 8.57 |
|  | **Moderate** | 5.71 |
|  | **Severe** | 2.86 |
|  | **Unable** | 0.00 |
| Usual activities | |  |
|  | **None** | 54.29 |
|  | **Slight** | 25.71 |
|  | **Moderate** | 11.43 |
|  | **Severe** | 8.57 |
|  | **Unable** | 0.00 |
| Pain/Discomfort | |  |
|  | **None** | 60.00 |
|  | **Slight** | 25.71 |
|  | **Moderate** | 8.57 |
|  | **Severe** | 5.71 |
|  | **Extreme** | 0.00 |
| Anxiety/Depression | |  |
|  | **None** | 71.43 |
|  | **Slight** | 25.71 |
|  | **Moderate** | 2.86 |
|  | **Severe** | 0.00 |
|  | **Extreme** | 0.00 |
|  |  |  |
| ^1^EQ-5D-5L Index | **Mean(SD)** | 0.84(0.26) |

^1^EQ-5D-5L responses are transformed using an algorithm into a single health state index score using the Irish value set. (Hobbins et al, 2018) . Completeness of data: 5% EQ-5D-5L missing

# Appendix 4: PRISMA: Pilot trial detail of patients receiving OAM from 09.01.2022 to 10.05.2022

67 patients attending ANP on 09.01.2022

37 patients approached whose assessment days were on the half-days available for the pilot location (space available 2/7 per week).

37 provided informed consent

n=37

## Identification

Completed course of treatment (n=2)

Stopped treatment prematurely due to toxicities/patient choice (n=1)

## Treatment course completed

n=34

4 admissions.

1 from virtual clinic and 2 from face-to-face clinics.

1 admitted from home mid- OAM cycle.

OAM paused during admission and recommenced on discharge (n=2)

Disease progression on admission. OAM stopped (n=2)

## Hospital admissions

n=32

## Disease progression

OAM switched to another OAM (n=2)

OAM stopped due to disease progression (n=3)

n=29

Disease progression on imaging) (n=5)

## End of trial

Continue with Oral Anti-Cancer Medications at end of trial (n=29)

1. Sláintecare is the ten-year programme to transform the Irish health and social care services. It is the roadmap for building a world-class health service aimed at care delivery closer to the patient’s home. [↑](#footnote-ref-1)
